# Increased Sensitivity to Thermal Pain Following a Single Opiate Dose Is Influenced by the COMT val^158^met Polymorphism

**DOI:** 10.1371/journal.pone.0006016

**Published:** 2009-06-23

**Authors:** Karin B. Jensen, Tina B. Lonsdorf, Martin Schalling, Eva Kosek, Martin Ingvar

**Affiliations:** 1 Osher Center For Integrative Medicine, Stockholm Brain Institute, Department of Clinical Neuroscience, Karolinska Institutet, Stockholm, Sweden; 2 Centre for Molecular Medicine, Karolinska Institutet, Stockholm, Sweden; Ohio State University Medical Center, United States of America

## Abstract

Increased pain sensitivity after opioid administration (opioid-induced hyperalgesia) and/or repeated painful stimuli is an individually varying and clinically important phenomenon. The functional polymorphism (val^158^met) of the Catechol-O-methyltransferase (COMT) gene regulates the metabolism of dopamine/noradrenaline. Individuals homozygous for the met^158^ allele have been reported to have increased pain sensitivity and there are findings of lower µ-opioid system activation during sustained pain. We hypothesized that met/met individuals would exhibit higher pain sensitization and opioid-induced hyperalgesia in response to repeated pain stimuli and an intravenous injection of an opioid drug.

Participants were 43 healthy subjects who went through an experiment where five blocks of pain were induced to the hand using a heat probe. After each stimulus subjects rated the pain on a visual analogue scale (VAS) from 0 mm (no pain) to 100 mm (worst possible pain). Before the second stimulus there was an intravenous injection of a rapid and potent opioid drug.

At baseline there was no difference in pain ratings between the COMTval^158^met genotypes, F(2, 39)<1. However, a repeated measures ANOVA for all five stimuli revealed a main effect for COMTval^158^met genotype, F(2, 36) = 4.17, p = 0.024. Met/met individuals reported significantly more pain compared to val/val, p = 0.010. A pairwise comparison of baseline and the opioid intervention demonstrated that analgesia was induced in all groups (p = 0.042) without a separating effect for genotype (n.s).

We suggest that the initial response of the descending pain system is not influenced by the COMTval^158^met polymorphism but when the system is challenged the difference is revealed. An important clinical implication of this may be that the COMTval^158^met related differences may be more expressed in individuals where the inhibitory system is already challenged and sensitive, e.g. chronic pain patients. This has to be proven in future studies where the impact of the COMTval^158^met polymorphism on opioid treatment in patients is addressed.

## Introduction

The subjective sensitivity to pain, as indicated by self-report in response to a pain stimulus, differs between individuals. Some are more sensitive than others and the subjective experience of pain is created from a complex interaction of afferent sensory input and cognitive processing of this stimulus [1]. The pain experience is modulated on all levels of the neural axis. Several brain networks act as potent modulators of pain and studies have shown that prefrontal brain regions are involved in the inhibition of nociception [2]. A suggested common pathway for these mechanisms is the recruitment of the descending pain defense system which is partly modulated by the central catecholaminergic systems [3], i.e. noradrenaline and dopamine. The function of these systems is genetically influenced by the activity of the catecholamine breakdown enzyme Catechol-O-methyltransferase (COMT) [4]. A single-nucleotide polymorphism (SNP) in the coding region of its gene (COMTval^158^met, rs4680) controls the enzyme activity and there are three possible genotypes of this polymorphism: met/met, met/val and val/val, representing the substitution of the amino acid valine for methionine at codon 158. The breakdown of dopamine and noradrenaline is up to 4 times higher for the valine allele compared to methionine, resulting in different levels of synaptic dopamine/noradrenalin following neurotransmitter release [4]. This polymorphism has previously been associated with aspects of memory function [5], anxiety [6] and regulation of pain sensitivity (7Individuals homozygous for the COMTmet^158^ allele have been reported to have increased pain sensitivity during sustained/repeated pain stimulation [7]–[8] but not following single pain stimuli [7], [9] compared to individuals homozygous for the COMTval^158^allele. This indicates that COMT influences central pain modulation. The latter is supported by findings of lower µ-opioid system activation during sustained pain stimulation in met/met individuals compared to val/val [8]. Furthermore, an increased µ-opioid receptor binding potential was found in certain brain regions in met/met individuals compared to val/val, which was interpreted as a compensatory mechanism [8]. This means that the COMTval^158^met polymorphism could affect the response to opioid drugs which was previously observed in a clinical setting including a group of cancer patients [10]–[13]. However, the relationship between the COMTval^158^met polymorphism and opioid analgesia was never investigated experimentally in humans. This is the first controlled experiment where the design aimed at clarifying the mechanisms behind a specific genetic influence on opioid analgesia in humans.

The endogenous opioid system is highly involved in endogenous regulation of pain [2] and therefore influences important aspects of what constitutes a pain response. The strong association between the COMTval^158^met polymorphism and prefrontal- and opioid function makes it a suitable candidate when investigating mechanisms underlying the placebo effect. Placebo analgesia involves biological mechanisms that recruit endogenous pain relief through strong influence on the subject's psychological expectancy and appraisal. When designing this study, we had much interest in addressing the genetic underpinnings for placebo analgesia. However, our experiment revealed that the genetic trait of the COMTval^158^met polymorphism had a profound effect on the conditioning step of the planned placebo intervention. This means that the opioid exposure introduced differences insensitivity to the following pain stimuli and thus confounding the original design. Here, we report on this effect as it represents an important step in understanding the mechanisms of pain sensitization and opioid-induced hyperalgesia.

We describe the association between the COMTval^158^met polymorphism and the analgesic effect of a short acting opioid on sensitivity to repeated pain stimuli in healthy subjects. Specifically, we investigate how the COMTval^158^met polymorphism influences the interindividual differences in response to intravenous injection of Remifentanil and placebo on repeated heat pain stimulation. Based on previous findings of increased µ-opioid receptor binding potential in met/met individuals, we would expect an increased analgesic effect of short acting opioids in met/met individuals compared to val/val. However, the reduced capacity to activate the µ-opioid system due to reduced concentrations of endogenous opioids [8] would predict increased pain sensitivity during repeated pain stimulation due to less effective recruitment of endogenous pain inhibition leading to a more pronounced sensitization in met/met individuals.

## Methods

Participants were 43 healthy subjects (12 men and 31 women, mean age 26 years), recruited via advertising. To meet the inclusion criteria subjects had to be over 18 years old, right-handed, take no medications (female subjects were allowed to take contraceptive pills), have no history of drug abuse, chronic pain or psychiatric disorders. Subjects were recruited from a variety of institutions in order to represent all sorts of educational and professional backgrounds. All subjects were Caucasian. The distribution of the different alleles of the COMTval^158^met polymorphism in the present sample and the sex distribution in the different genetic groups are seen in Table 1. The genotype distribution for COMTval^158^met in this sample did not differ significantly from the one predicted by the Hardy-Weinberg equilibrium, *χ2* = 0.183, p = 0.67.

**Table 1:** Distribution of the different COMTval^158^met polymorphisms for male and female subjects in the present study (n = 43).

Subjects	COMT		
	met/met	met/val	val/val
Male	5	5	2
Female	5	15	11
Total	10	20	13

This study was conducted according to the principles expressed in the Declaration of Helsinki. The study was approved by the Institutional Review Board of Karolinska Institutet (Reference number 2005/950–31/1). All patients provided written informed consent for the collection of samples and subsequent analysis.

The subjects went through an experiment where pain was repeatedly induced to different locations of the dorsum of the hand using a 3×3 cm heat probe (Medoc TSA, Medoc, Israel). Hence, no part of the skin was affected by the heat probe more than once as to prevent increased sensitivity to the pain stimulus by means of local mechanisms. The order of the different positions on the hand was counter-balanced and in total five blocks (T1–T5) of 30 seconds of tonic heat (48° Celsius) was administered. After each stimulus T (T1–T5) subjects were asked to rate the pain on a visual analogue scale (VAS) ranging from 0 mm (no pain) to 100 mm (worst possible pain). There was at least a 10 minutes pause between all pain stimuli. Only one block (T2) included an active drug treatment. Immediately before T2 there was an intravenous injection of a rapid and potent anesthetic drug Remifentanil (4-(Methoxycarbonyl)-4-[(1-oxopropyl) phenylamino]-1 - piperidine propa-noic acid methyl ester) which is an opioid drug acting on µ-type receptors. It has a rapid onset of action and a very short duration. Patients were informed that the drug used in the experiment had a very short time of action and that the effect would last only for the duration of the pain stimulation. The time for a 50% reduction in the effect site concentration is about 3.65 min and the terminal elimination half-life 10.2 min at a dose of 2 g/kg. The dose used in our experiment (0.08 µg/kg body weight) was dramatically smaller than in the given example but we still employed a 1 hour pause before the next stimulus (T3) in order to minimize a possible residual drug effect. The optimal dose of Remifentanil was determined in a pilot study where the desired analgesic effect was validated. At T4 there was an intravenous injection of a placebo substance (saline) together with the instruction that also this injection included an anesthetic. All other pain stimuli in the experiment had no concomitant treatment or oral suggestions. During the experiment both the subject and experimentor were blinded as to the genetic category of the subject. The experiment lasted for approximately two hours.

Samples of 20 ml whole blood were taken venously and stored at −80°C until DNA extraction, which was performed robotized by the local Biobank (KI Biobank, Karolinska Institutet, Stockholm) using standard methods (Autopure LS system, Gentra Systems, Minneapolis, MN). DNA yield was measured with UV at 260 nm and with the 260/280 ratio as a quality check. For genotyping of COMTval^158^met (rs4680) a commercial TaqMan® assay was used. Fluorescence measurements were performed using the ABI HT7900 (Applied Biosystems, Foster City, CA) and the SDS 2.2.1 software (Applied Biosystems). All genotypes were determined twice.

Firstly, we probed for a difference in pain response based on COMTval^158^met genotypes for the first exposure to heat pain by means of a univariate ANOVA (dependent variable VAS ratings, fixed factor COMTval^158^met genotype).

The VAS ratings were normalized to the initial baseline rating, i.e. each participant's T2, T3, T4 and T5 rating was individually divided by the participant's baseline rating. This is a way to control the effect of individual differences in response style, i.e. answering in the high end or low end of the VAS scale when reporting the subjective level of pain.

For the main analysis a repeated measures ANOVA was performed where the normalized pain responses for T1–T5 were used as within-subject factor and the three different COMTval158met genotypes were used as between-subject factors.

Thereafter, we probed for an overall effect of time (T), as well as a COMTval^158^met related difference in the analgesic response to Remifentanil by means of pairwise comparisons derived from the above model.

To explore the main effects and a possible interaction for the three provocations in the post Remifentanil period a repeated measures ANOVA was perfomed for T3–T5 (between subjects factor was COMTval^158^met genotype, within subjects factor was the three different timepoints).

In order to control for any possible differences in pain ratings between men and women, sex was added as covariate in the analysis.

In all performed analyses Greenhouse Geisser corrections were applied when appropriate. The statistical software SPSS 16.0 was used. Hardy-Weinberg equilibrium was calculated by Pearson's goodness-of-fit *χ2* (df = 1) using a website (http://ihg2.helmholtz-muenchen.de/cgi-bin/hw/hwa1.pl).

## Results

At baseline (T1) there was no difference in pain ratings between the COMTval^158^met genotype groups, F(2, 40)<1, p = 0.741.

**Figure 1:**
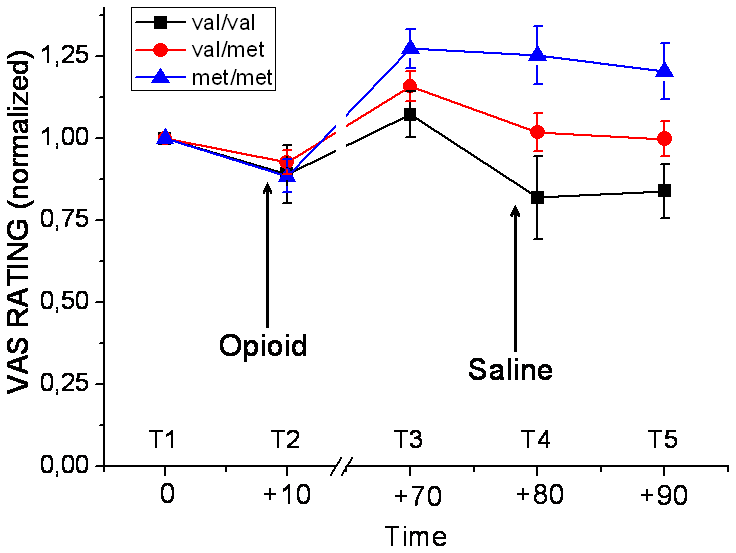
Representation of the subjects' pain ratings over the five different time points (T). Pain was rated using the visual analogue scale (VAS) ranging from “no pain” (0 mm VAS) up to “worst possible pain” (100 mm VAS). Identical pain stimulation was given at all five timepoints. Prior to T2 there was an intravenous injection of Remifentanil and prior to T4 there was an intravenous injection of saline (placebo). All pain ratings represented in this figure are individually normalized to baseline (T1).

When looking at the pain reports over all five timepoints (T1–T5) for COMTval^158^met (T×COMT), a repeated measurements ANOVA revealed a main effect for COMTval^158^met genotype, F(2, 36) = 4.17, p = 0.024 (see Figure 1). Individuals homozygous for the met allele reported significantly more pain as compared to individuals homozygous for the val allele, p = 0.010 and also heterozygous, p = 0.042. Carriers of one or two val alleles did not differ in their pain reports, p = 0.265. The main effect for T was significant, F(3,109) = 5.70, p = 0.001. There was a significant interaction for T×COMT, F(6, 109) = 3.38, p = 0.004. For the total experiment there was no main effect of sex, F(1,36)<1, p = 0.128, or interaction of T×sex, F(3,109)<1, p = 0.329.

A pairwise comparison for T1–T2 demonstrated that analgesia was induced by Remifentanil (T2) in all groups without separating the different genotype groups (p = 0.042). This means that there was no significant effect of genotype on the direct analgesic response to the opioid.

In the post Remifentanil period (T3–T5) there was a significant difference between the COMTval^158^met genotype groups, F(2,36) = 6.48, p = 0.004, with highest VAS ratings given by the met/met group and lowest in the val/val with the heterozygous in between. Individuals homozygous for the met allele reported significantly more pain than individuals homozygous for the val allele, p = 0.002, and heterozygous, p = 0.014, both of which did not differ from each other. There was also a main effect for T with decreasing VAS reported with T, F(2,64) = 5.39, p = 0.009. However there was no interaction T×COMT F(4,64)<1, p = 0.708. Thus, there was no effect of the placebo manipulation at T4 related to genotype group. There was no main effect of sex for the post Remifentanil period, F(1,36) = 2.67, p = 0.11, or interaction of T×sex, F(2,64) = 0.73, p = 0.47.

## Discussion

Our data demonstrate that the COMT val^158^met genotype has a marked influence on the pain experience following standard heat pain provocations in the period following an opiate injection. Also, several other findings should be pointed out: We found that the reaction to the initial pain stimulus was the same for individuals with different COMTval^158^met genotypes. This is in concert with the report of Kim et al (2004) as well as Diatchenko et al (2006) that used a repeated pain provocation design. In that report, there were no differences based on COMTval^158^met genotypes for the initial pain provocations, but a temporal heat pain summation was noted that differed between genotypes, with the most expressed summation expressed in the met/met group. In that study a fast repeat of the stimuli was used and also the same anatomical site for stimulation was employed in all cases. In our study we used different anatomical locations and had minimally 10 min between stimuli and a one hour washout of Remifentanil between T2 and T3. We therefore do not see the data as directly comparable except for the initial time point. The lack of influence of COMTval^158^met genotypes in the analgesic response to the intravenous opioid administration is important. It suggests that the initial response of the pain system is not influenced by the COMTval^158^met polymorphism but when the pain defense system is challenged repeatedly, this specific genotype based difference become apparent. Such a vulnerability effect has previously been described for the COMTval^158^met polymorphism in the effect on working memory in relation to aging [14]. A possible clinical implication of this may be that the COMTval^158^met related differences may be more expressed in individuals where the inhibitory system is already challenged and the individual is sensitive already from start, e.g. chronic pain patients.

When we analyzed all five stimulations in the experiment, there was a difference in pain response for individuals with the different COMTval^158^met genotypes. The repeated measures ANOVA revealed main effects for T as well as COMT genotype and also a significant interaction. Further analyses revealed that the main effect for COMTval^158^met genotype was driven by the three post Remifentanil provocations (T3–T5), which demonstrated a consistent effect for COMTval^158^met with no interaction with T. The met/met group exhibited the highest pain ratings, the val/val group the lowest and the heterozygous group had an intermediate response. Due to the notable effect of the COMTval^158^met polymorphism on pain sensitivity over time, it was not possible to detect an effect of the placebo manipulation at T4. The factor “sex” was always added as covariate in order to control for the possibility that it could be a confounding factor, explaining differences in pain sensitivity. Our conclusion from the statistical analysis is that the influence of COMTval^158^met on pain sensitivity is not affected by sex. However, the distribution between the different genotypes was not the same for males and females in this study, limiting the possibility to fully interpret the impact of COMTval^158^met between men and women.

The increased pain sensitivity for the met/met individuals following Remifentanil could be caused by reduced efficacy of endogenous pain modulation and/or by increased susceptibility to opioid-induced hyperalgesia. Opioid-induced hyperalgesia has been reported following a single intravenous infusion of Remifentanil in humans [5]. The mechanisms involved in opioid-induced hyperalgesia overlap to a large degree with those implicated in pain sensitization following repeated or tonic noxious stimuli such as activation of N-methyl-D-aspartate (NMDA) receptors and activation of descending pain facilitatory mechanisms [15]–[18]. The design of our study does not permit a definite distinction between pain sensitization due to opioid exposure or repeated painful stimulation. In both cases the result is increased pain sensitivity, but the mechanisms leading to this are different. However, the fact that the post Remifentanil pain ratings decreased over time favor opioid induced hyperalgesia as a likely explanation. If our results would be due to a sensitization through repeated pain stimuli there would probably be a continuous increase up to T5. Our results therefore suggest that the COMTval^158^met polymorphism could be important for the understanding of why certain individuals are more prone to develop opioid-induced hyperalgesia and tolerance to the antinociceptive actions of opioids.

Regardless of the possible impact of COMTval^158^met on pain sensitization and/or opioid-induced hyperalgesia, results from the present study support the hypothesis that the COMTval1^58^met polymorphism may influence the capacity for top-down regulation of pain. The COMTval^158^met polymorphism is a key regulator of dopaminergic neurotransmission and has consistently been associated with human prefrontal function. Studies show that the effect of different COMTval^158^met genotypes is stronger the more it involves frontal cortical structures tuned by dopamine [5]. The prefrontal cortex of the human brain is a key modulator for descending inhibition of pain, which is partially mediated by endogenous opioids and catecholaminergic mechanisms. These mechanisms are triggered by repeated or tonic noxious stimuli, which mean that the inhibitory effect kicks in only after a proper activation of the pain suppressing system.

In a study by Zubieta et al. (2003) there was a significantly reduced µ-opioid response in the brain during pain in met/met individuals in response to a tonic pain model where hypertonic saline was continuously infused in the masseter muscle. There was also an observation of differences between groups in the quantities of hypertonic saline needed to maintain a stable pain level over time, indicating that the met/met group needed less hypertonic saline. The results from our present study validate the findings by Zubieta et al (2003) by demonstrating increased pain sensitivity in met/met individuals over time also following administration of exogenous opioids.

The opioid injection given at T2 led to significant reduction in pain, but there was no difference in effect between the three different genotype groups. This negative result was different from our hypothesis. We expected that there would be an increased analgesic effect in met carriers compared to val/val since there are reports of increased µ-opioid receptor binding potentials and possibly a decreased ability to recruit endogenous opioids. Hence, the negative result for differences in opioid response suggests that there is no difference in an initial opioid effect. However, with repeated painful stimuli following this single opioid administration the mechanisms that are associated with the COMT val^158^met polymorphism can be manifested. In the study by Zubieta et al (2003) the correlation between pain sensitivity and genotype was seen first after a prolonged pain stressor. Results from the present study validate this also in response to exogenous opioids. Previous results on the influence of the COMTval^158^met polymorphism in opioid treatment in a group of cancer patients [10]–[13] showed that met/met individuals needed lower doses of morphine in order to relieve pain. That can partly be explained by increased µ-opioid receptor binding potentials and possibly a decreased ability to recruit endogenous opioids in met/met individuals [8].

Originally there was also an intention to analyze the difference in placebo response between the different COMTval^158^met genotypes. Instead the notable difference in pain sensitivity that developed over time for the different COMTval^158^met genotypes was discussed *per se*. This leaves the placebo questions for future studies with experimental designs that are not confounded by the difference in pain sensitivity after an opioid injection and repeated pain stimuli.

The choice of genetic polymorphism was based on the empirical evidence for the involvement of COMTval^158^met in regulation of pain, which allowed us to create a strong a priori hypothesis. Compared to other possible choices, this is a functional polymorphism which means it has a well documented impact on physiological processes, and in particular the enzyme activity of COMT. Also, the frequency of the different alleles in this particular polymorphism is fairly equal in distribution which makes it a good candidate when one does not have access to a large group of genotyped subjects. The relatively small number of subjects included in this experiment is a limitation to the study. However, this is the first experimental study on the COMTval^158^met influence on opioid anesthesia and the results could potentially be of high scientific and clinical benefit. Including a small group of subjects means a greater risk for false positives and the results should be carefully interpreted. Therefore we hope for validation of these results in future studies with more resources and subjects.

Here we demonstrate that the response dynamics following opioid administration and repeated pain stimulation can be stratified based on genotype. This emphasizes the complexity of the mechanisms involved in pain inhibition.
